# Dynamic Characterization of the Human Heme Nitric Oxide/Oxygen (HNOX) Domain under the Influence of Diatomic Gaseous Ligands

**DOI:** 10.3390/ijms20030698

**Published:** 2019-02-06

**Authors:** Rana Rehan Khalid, Abdul Rauf Siddiqi, Efstratios Mylonas, Arooma Maryam, Michael Kokkinidis

**Affiliations:** 1 Department of Biosciences, COMSATS University, Islamabad 45550, Pakistan; ray.binm@gmail.com (R.R.K.); aroomabinm@yahoo.com (A.M.); 2 Department of Biology, University of Crete, 70013 Heraklion, Greece; 3 Institute of Molecular Biology and Biotechnology, Foundation for Research and Technology-Hellas (IMBB-FORTH), 70013 Heraklion, Greece; stratos_mylonas@imbb.forth.gr

**Keywords:** quantum calculations, soluble guanylate cyclase, molecular dynamics simulations, metal center parameter builder, hydrogen bond occupancy, principal component analysis

## Abstract

Soluble guanylate cyclase (sGC) regulates numerous physiological processes. The β subunit Heme Nitric Oxide/Oxygen (HNOX) domain makes this protein sensitive to small gaseous ligands. The structural basis of the activation mechanism of sGC under the influence of ligands (NO, O_2_, CO) is poorly understood. We examine the effect of different ligands on the human sGC HNOX domain. HNOX systems with gaseous ligands were generated and explored using Molecular Dynamics (MD). The distance between heme Fe^2+^ and histidine in the NO-ligated HNOX (NO-HNOX) system is larger compared to the O_2_, CO systems. NO-HNOX rapidly adopts the conformation of the five-group metal coordination system. Loops α, β, γ and helix-f exhibit increased mobility and different hydrogen bond networks in NO-HNOX compared to the other systems. The removal of His from the Fe coordination sphere in NO-HNOX is assisted by interaction of the imidazole ring with the surrounding residues which in turn leads to the release of signaling helix-f and activation of the sGC enzyme. Insights into the conformational dynamics of a human sGC HNOX domain, especially for regions which are functionally critical for signal transduction, are valuable in the understanding of cardiovascular diseases.

## 1. Introduction

There are two types of enzymes with guanylyl cyclase activity; membrane-bound particulate guanylate cyclase (pGC) and cytosolic soluble guanylate cyclase (sGC) [1]. These enzymes are crucial for cGMP production, which regulates numerous physiological and pathophysiological processes [2], such as cardiovascular diseases, platelet aggregation, neurodegeneration, erectile dysfunction, etc. Knowledge of the structure and dynamics of the sGC enzyme is indispensable to understanding the mechanism of function [3]. Many studies have shown that the Heme Nitric Oxide/Oxygen (HNOX) domain of sGC is the starting point of signal transduction. The heme prosthetic group allows this domain to sense the small gaseous ligands CO, O_2,_ and NO [4]. It is the binding of NO to the HNOX domain that stimulates the catalytic domain to cyclize GTP into cGMP, responsible for regulating many downstream pathways. sGC is a 150 kDa macromolecule consisting of subunits α and β [5,6]. The complete structure of sGC has not yet been elucidated, and so therefore, the interdomain interactions of sGC remain poorly understood. Many aspects of the structure and dynamics of the protein and its individual domains still require a detailed and comprehensive analysis.

The crystal structure of the bacterial HNOX domain has been resolved, but essential heme binding pocket residues (Y135, S137, and R139, Y-S-R motif) are also conserved in the human HNOX [7]. In this domain diatomic gaseous molecules such as NO, CO, and O_2_ bind with the heme group on the so-called distal site, while on the proximal side a coordinate bond forms between heme and histidine 105 (H105) (6c) [8]. The heme group contains propionic acid moieties involved in hydrogen bond interactions with the conserved distal pocket residues of the Y-S-R motif [9]. The reduced heme of sGC favors the binding of diatomic gas molecules, notably CO and NO [10]. Heme is found inside the HNOX domain in two coordination states, i.e., a six coordination (6c) and a five coordination (5c) state. During activation, the gaseous ligand NO binds to heme and changes its state from 6c to 5c. Breaking of the coordinate bond between H105 and iron results in the activation of sGC [11,12,13].

Dealing with metalloproteins like sGC poses significant challenges. Iron (Fe) is a transition metal and can exist in ferrous +2 and ferric +3 states. The Fe^2+^ coordination state of the HNOX heme favors the binding of small gaseous ligands, such as NO, O_2_, and CO [14]. The affinity of NO and CO ligands for the heme prosthetic group of sGC is considerably higher than the affinity of O_2_. Discrimination between these diatomic molecules is controlled by heme distal site residues, whereas residues surrounding the proximal histidine perturb the electron density of the imidazole ring, further contributing to the transfer of charge from histidine to Fe^2+^. When NO binds to 5c heme, the d orbital electrons of the iron move to an unstable high spin state, with the formation of a transient 6c complex, that converts to 5c with the loss of the histidine-iron interaction [15,16].

In this paper, we elucidate the atomistic dynamics of NO/O_2_/CO-ligated human HNOX domain in comparison with control apo-HNOX. A comparative modeling approach was used to estimate the structure of the human HNOX domain. The models were then further subjected to detailed dynamics investigation. Stability and hydrogen bond analysis revealed the binding pocket structural transitions in response to different ligands (NO, O_2_, CO). Furthermore, essential dynamics (ED) were employed to identify noticeable structural variations. The following five complexes were examined; 1. Apo-HNOX (without gaseous ligands), 2. NO-HNOX (5c) 3. NO-HNOX (6c) with the bond between H105 and Fe removed to study the effects of bond-breaking (5c), 4. O_2_-HNOX (6c) 5. CO-HNOX (6c). Each system is examined for 50 ns.

## 2. Results and Discussion

### 2.1. Homology Modeling

**Figure 1:**
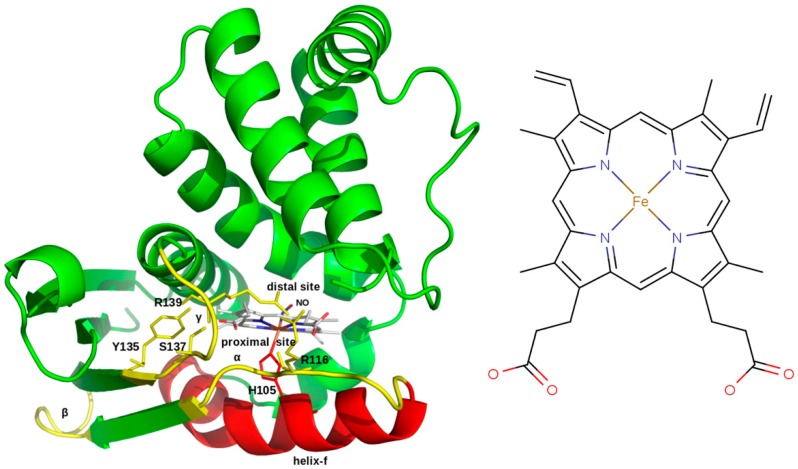
Predicted model of the 6c coordination state of the Human HNOX domain where the binding pocket heme is ligated to NO (6c NO-HNOX). The Y135-S137-R139 (Y-S-R) motif that bears strong interactions with the heme propionic moiety is also labeled (yellow). Functionally critical regions are also labeled as helix-f (red), loop α, β, and γ (yellow). A simple chemical scheme of heme is shown in the right panel. A detailed scheme of the heme interactions for each system are shown in Figure 7.

The HNOX domain harbors a heme prosthetic group which can sense diatomic gaseous molecules such as CO, NO, and O_2_. The human HNOX N-terminal domain (1–186 a.a) has 34% sequence identity with the nostoc bacterial template. Modeling results showed that 94.6% of the residues are found in favored regions of the Ramachandran plot and 97.8% in allowed regions. After energy minimization through YASARA (Figure 1) 98.4% of residues occupied favored regions and 99.5% allowed regions. The global structure quality score [17] of the predicted model is statistically significant (TM = 0.83), which makes the model reliable for further structural interpretation.

### 2.2. QM Calculations

**Figure 2:**
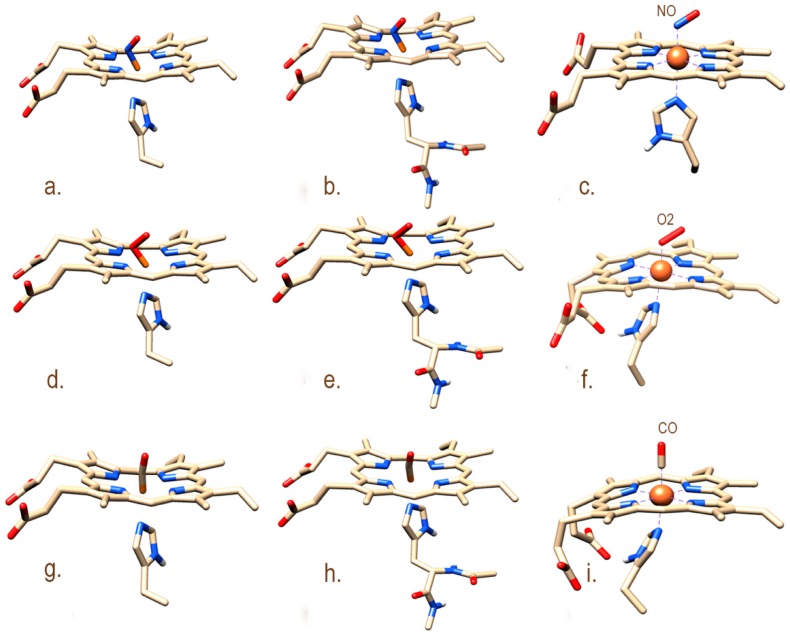
HNOX active site models used in the parametrization procedure. (**a**,**b**), (**d**,**e**), and (**g**,**h**) represent the small and large model systems of active sites His-heme-NO, His-heme-O_2_ and His-heme-CO, respectively. Panels (**c**,**f**,**i**) show the optimized active site of the smaller models (**a**,**d**,**g**) that was further used for the parameterization of His-heme-NO/O_2_/CO.

The Metal Center Parameter Builder (MCPB.py) was used in conjunction with Gaussian09 to parametrize the active site (His-heme-NO/O_2_/CO) using models of differing sizes to balance speed and accuracy [18] (Figure 2). Smaller models were used to estimate the iron associated bond and angle parameters, while the larger models were used for partial charge calculations. The accuracy of the optimized small model metal-associated bonds and angles was evaluated with Chimera [19]. The coordination states of aforesaid systems remain stable during optimization and force constant calculations (Figure 2c,f,i). The same level of theory and the same basis set (B3LYP/6-31G*) was applied to all complexes to ensure local minima were found. The force constants of the active site atoms were estimated with the Seminario method [20] and all bonded and non-bonded type parameters were within acceptable range. The histidine imidazole ring adopts a stagger conformation forming a 45° angle with the plane of heme opposite to pyrrolic nitrogen atoms. This conformation is favorable to the binding of gaseous ligand such as O_2_. Furthermore, the distance between the histidine imidazole ring and iron is high, in the NO-ligated system, in contrast to the CO and O_2_ ones. Moreover, O_2_- and NO-bonded complexes have bent conformations; however, the CO-ligated complex adopts a linear configuration with a 179° angle (Figure 2c,f,i).

### 2.3. MD Stability Analysis

**Figure 3:**
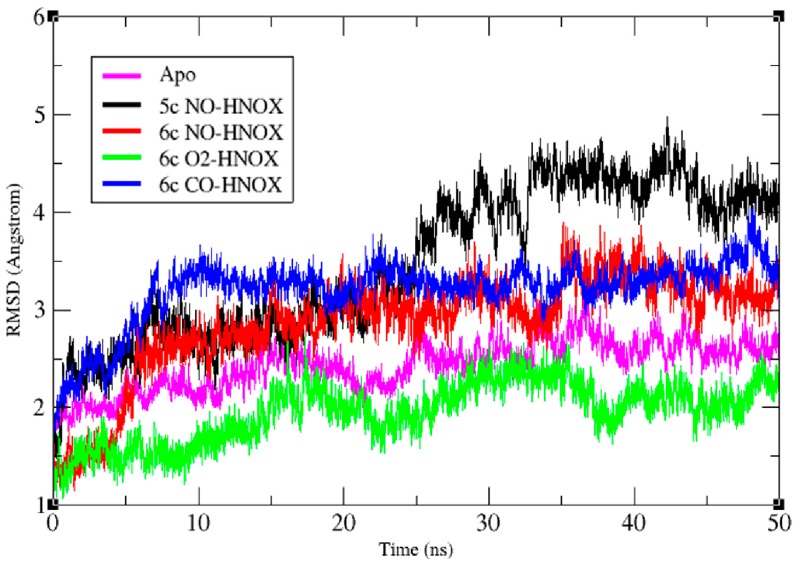
Time-dependent RMSD plot of the backbone atoms of apo, 5c NO-HNOX, 6c NO-HNOX, and 6c O_2_/CO-HNOX. Magenta, black, red, green and blue represent the RMSD plots of apo, 5c NO-HNOX, 6c NO-HNOX, 6c O_2_-HNOX and 6c CO-HNOX, respectively.

CPPTRAJ [21] was used to evaluate the conformational stability of the HNOX systems over the course of the simulation. The root-mean-square deviation (RMSD) of the five systems; apo-HNOX, 6c NO/CO/O_2−_ HNOX and 5c NO-HNOX, was analyzed for all backbone atoms, and the RMSD results indicate that the largest variations were observed in the NO/CO-HNOX systems in contrast to the O_2_ and apo system that did not experience noteworthy RMSD variations (Figure 3). In the 6c NO-HNOX system the Fe-H105 bond length increased abruptly at the time interval of 4–6 ns. This is possibly the point at which the activation of the HNOX domain occurs with the loss of the Fe-H105 interaction. Both NO-bonded HNOX complexes exhibit an average RMSD of 2.7 Å in the time interval of 6–25 ns while from 25 ns to 50 ns the average RMSD of 5c NO-HNOX was further increased to 4.4 Å which can be attributed to the release of signaling helix-f. Of the other systems, the CO-ligated one underwent the highest RMSD fluctuations with an average RMSD of 3.4 Å. The loops α, β and the flanking helix-f were the regions which contributed the most to the RMSD increase. 

**Figure 4:**
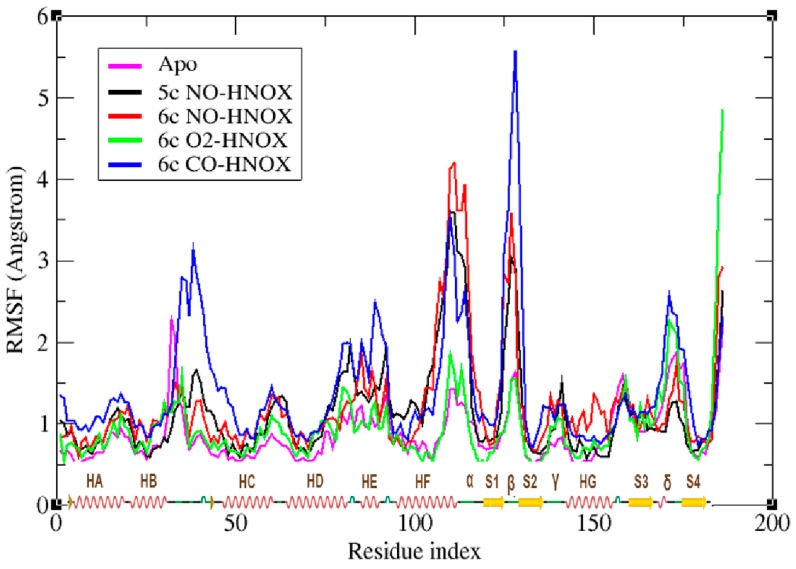
The plot depicts the residual fluctuations of human HNOX domain (1–186 a.a.) under the influence of gaseous ligands NO, O_2_ and CO. The x-axis shows the residue number, as well as the secondary structure elements of the H-NOX domain; helices are represented as red springs (HA, HB, HC, HD, HE, HF, HG), loops as green lines (α, β, γ, δ), and strands as yellow arrows (S1, S2, S3, S4). The y-axis represents the residual mobility around its mean position. Magenta, black, red, green, and blue represent the RMSD plots of apo, 5c NO-HNOX, 6c NO-HNOX, 6c O_2_-HNOX and 6c CO-HNOX, respectively.

Root mean square fluctuation (RMSF) stability analysis helps to investigate the mobility of residues under the influence of different ligands over the course of the entire simulation. The peaks represent areas of increased residual flexibility (Figure 4). The results indicate that O_2_ binding to the HNOX domain has minor effects on the residual fluctuations, similar to apo-HNOX. In NO-bonded complexes regions 105–118 (helix-f, loop α) and 124-129 (loop β) are significantly more mobile with a maximum RMSF of 4.2 Å for loop α. The 5c NO-HNOX system exhibited a similar behavior with a slightly smaller peak of 3.6 Å. In contrast, the highest peak of the CO-ligated system at 5.1 Å is observed at the loop β region. These observations suggest that the aforementioned sites possibly contribute to the release of heme from H105.

**Figure 5:**
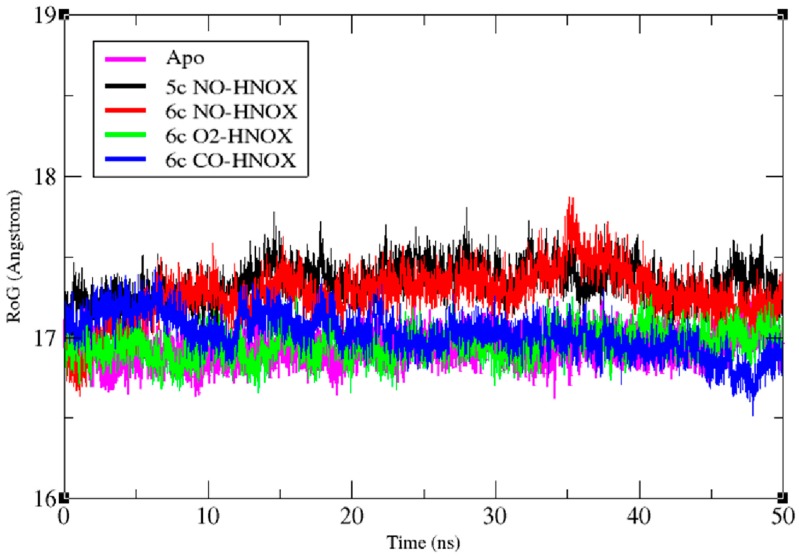
Time-dependent plot of the radius of gyration (R_g_) for apo, 6c NO/CO/O_2_-HNOX and 5c NO-HNOX systems. Magenta, black, red, green and blue represent the plots of apo, 5c NO-HNOX, 6c NO-HNOX, 6c O_2_-HNOX, and 6c CO-HNOX, respectively.

Another conformational stability indicator, the radius of gyration (R_g_), provides a measure of the compactness of the protein by monitoring the effective size of protein. R_g_ is defined as the mass-weighted root mean square distance of a group of atoms from their common center of mass (Figure 5). The R_g_-analysis results are consistent with previous stability findings. The O_2_-ligated 6c HNOX radius of gyration remains stable at ~16.9 Å. The 5c NO-HNOX system has the highest average R_g_ at 17.4 Å. The broadest range of configurational space variation was observed for the 6c NO-HNOX system with an R_g_ below 17 Å at the beginning of the simulation, stabilizing at ~17.3 Å after 6ns. Finally, the average R_g_ of apo and CO-ligated systems was ~17 Å.

### 2.4. Hydrogen Bond Occurrence

**Figure 6:**
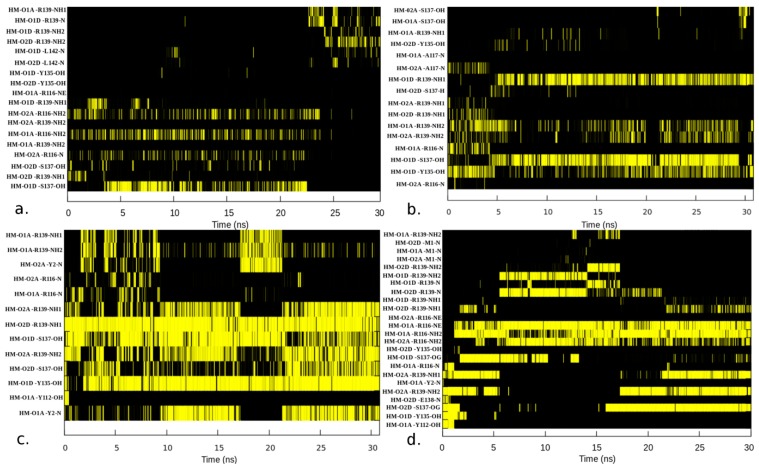
Binding modes of (**a**) 5c NO-HNOX, (**b**) 6c NO-HNOX, (**c**) O_2_-HNOX and (**d**) CO-HNOX complexes with heme carboxyl groups. The x-axis represents the time (ns) while the y-axis shows the heme (HM) propionic moiety (O1A, O2A, O1D, O2D) and critical binding pocket residues atoms interacting by hydrogen bonds. Yellow color illustrates the presence of hydrogen bond while black color represents absence.

Hydrogen bond analysis was performed to elucidate the binding modes of 6c O_2_/NO/CO-HNOX and 5c NO-HNOX systems (Figure 6). Although the number of O_2_-HNOX binding pocket residues that take part in electrostatic interactions with heme is smaller, they show a more robust hydrogen bond network compared to the NO and CO complexes. The binding modes pattern of apo-HNOX (not shown) is very similar to the O_2_-HNOX, except for the Y2 and R116 electrostatic interactions. The hydrogen bond fractional occurrence is high because the O_2_-HNOX (and apo-HNOX) system does not undergo as drastic conformational transitions as the NO/CO-bonded systems. Residues Y135, R137, R139, R116, and H105 are the major contributors of hydrogen bonds with the heme propionic acid moiety in all four systems. Weaker hydrogen bond interactions were also found for M1, Y49, A117, E138, and L142 in the different systems (Table 1). H105 hydrogen donor residues such as A109, L108, and acceptor residues such as 101L, 102A, and 118P may be involved in the perturbation of the imidazole ring electron density leading to the formation of the 5c heme-NO complex (Table 2). Earlier studies suggest that the interaction of H105 with the surrounding residues drive the imidazole ring to a high spin state, while the slightly negative charge of NO favors the low spin state, resulting in stretching of the bond between iron (Fe^2+^) and H105 [22,23,24]. Furthermore, interaction of R116 with the heme carboxylic groups (O1A, O2A, O1D, O2D) is stronger in 5c NO-HNOX and 6c CO-HNOX complexes, compared to the apo, 6c O_2_-HNOX and 6c NO-HNOX complexes (Table 1, Figure 6). The liberation of the heme cofactor from H105 might be contributing to increasing R116 hydrogen bonding with the heme carboxylic groups, compared to 6c NO-HNOX. This interaction possibly enhances the structural fluctuations of loop α, further contributing to sGC activation. Previous studies have shown that residue R116 resides on the periphery of HNOX, facing towards the adjacent PAS domain [25], implicating it in cyclase activity. Moreover, the apo-HNOX and O_2_-HNOX complexes heme propionic moiety bears considerable hydrogen bond interactions with Y2, Y112, and Y135 enhancing the overall compactness of the system (Table 1, Figure 6). It is also discerned that heme carboxyl group in CO-HNOX showed more robust interaction with R116 amongst all four systems, although there is no H105-Fe bond cleavage observed in CO-HNOX system (Table 1).

**Figure 7:**
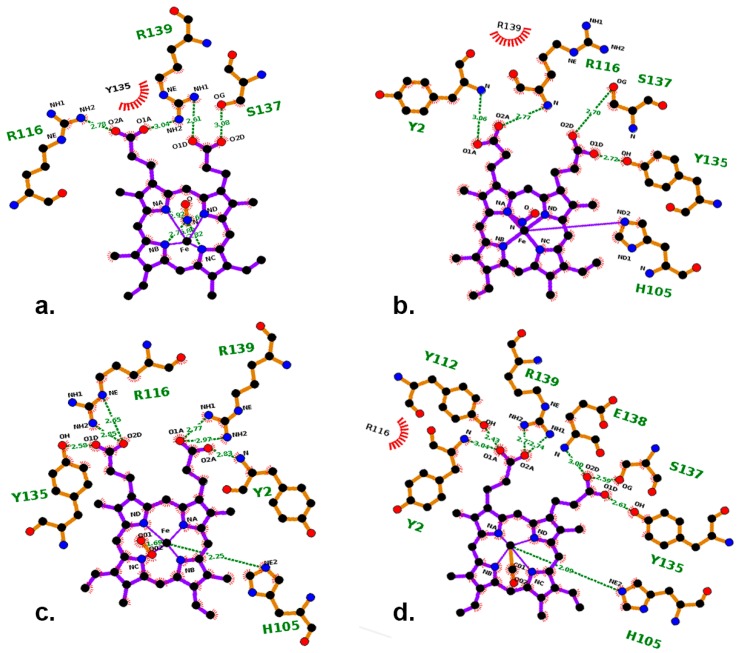
(**a**–**d**) represents the non-bonded interactions of 5c NO-HNOX, 6c NO-HNOX, O_2_-HNOX and CO-HNOX systems respectively. The propionic moiety of heme (O1A, O2A, O1D, O2D) prominently interacts with functionally important residues such as Y112, Y135, S137, R139, R116 and Y2 throughout the simulation.

To visualize the entirety of the electrostatic interactions of heme with the diatomic ligands (NO, CO, O_2_), all systems were treated with the Ligplot tool [26]. The H105-Fe bond undergoes a bond stretching of 3.23 Å in the NO-ligated system (Figure 7) contributing to the release of signaling helix-f while establishing a robust interaction with R116. In contrast, the bond length remained less than 2.5 Å in the CO_−_ and O_2_-ligated systems.

### 2.5. Essential Dynamics

Essential dynamics was performed to identify the overall normalized pattern of motion in all four systems. The principal component analysis facilitates further elucidation of the sGC activation mechanism. The diagonalization of the covariance matrix contributes to getting more meaningful configurational space, revealing functionally critical structural transitions [27]. The backbone atoms of apo, CO/NO/O_2_-HNOX complexes were considered to perform essential dynamics. Interestingly, the first two principal components correspond to 85% of the overall positional transitions in all systems. The motion of the first two dominant modes was examined through the VMD normal mode analysis plugin and the porcupine plot was used to show the magnitude and direction of selected eigenvectors [28]. 

**Figure 8:**
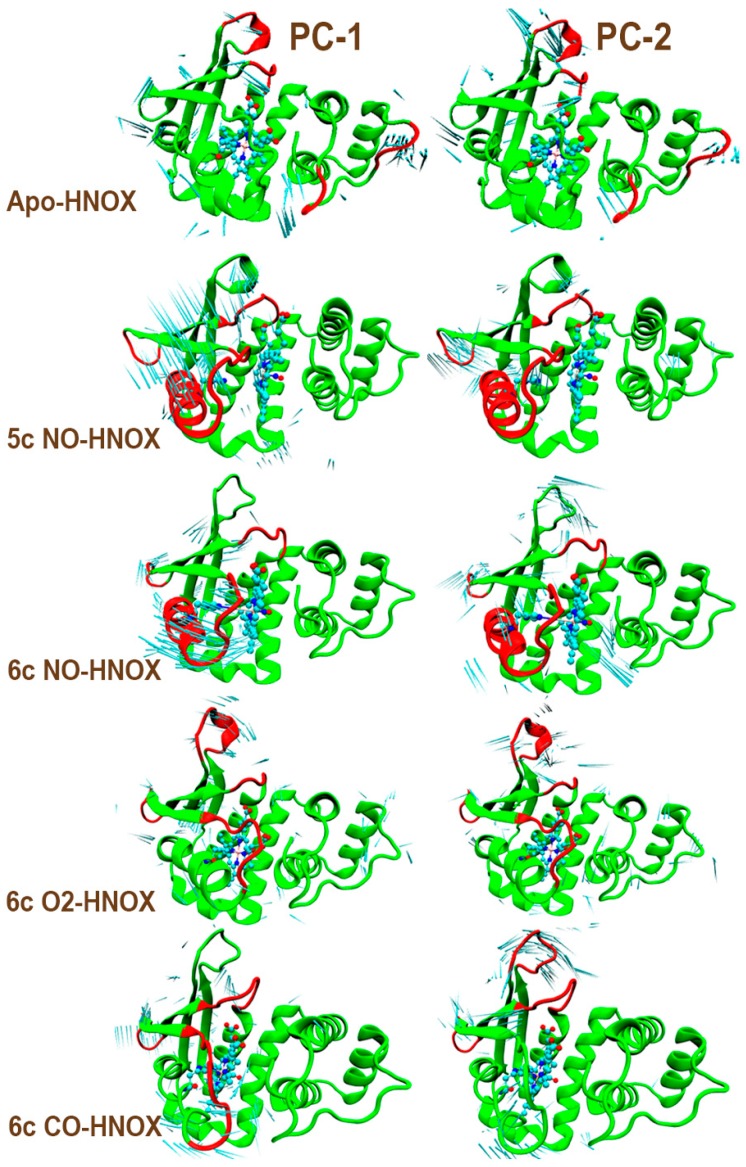
Porcupine plot illustrating the highest principal component variance of the first (PC-1) and second (PC-2) modes of motion. Highly fluctuating regions are highlighted in red. The length of the cyan “needles” represents the degree of mobility.

**Figure 9:**
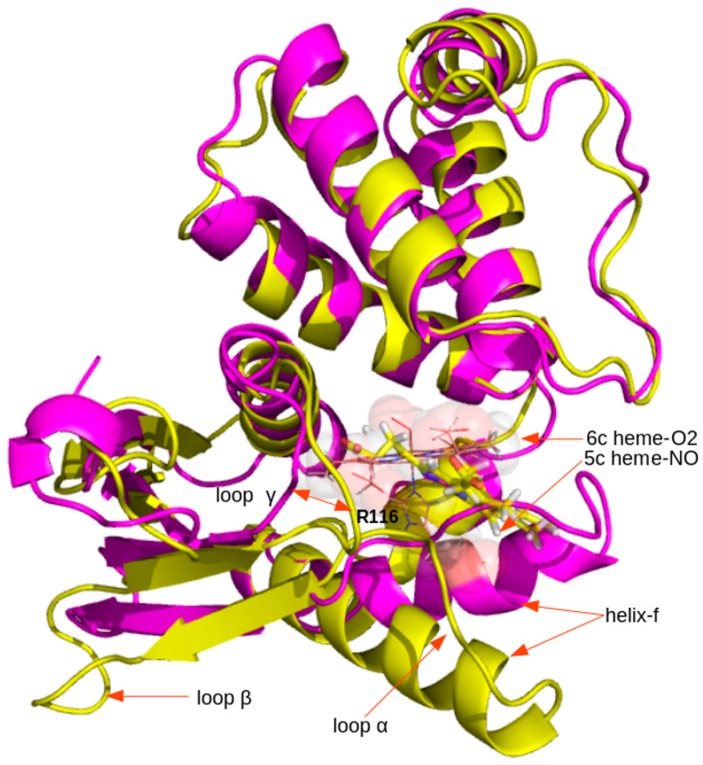
Superposition of HNOX-NO (yellow) and O_2_ (magenta) systems. The heme-O_2_ ligand is shown as transparent spheres while the heme-NO ligand is shown as sticks. Regions assumed to be important for signal transduction (helix-f, loops α, β and γ) experienced noticeable structural changes after ligation of NO to HNOX.

Critical regions of the human HNOX domain that contribute to high variance during essential dynamic analysis are shown in Figure 8. The regions include helix-f and loop α and β in the NO-ligated HNOX complexes, although in the case of the CO-ligated complex the helix-f displacement was much less pronounced. Interestingly, in the control simulation (apo-HNOX), helix-f and loop α and β regions experienced trivial structural transitions, in contrast with other systems. These results are compatible with the stability analysis. Moreover, the length of the Fe-H105 bond strongly fluctuates in the 6c NO-HNOX system. Experimental data [11] has shown that the Fe-H105 bond breaking event upon NO binding to heme, during the sGC activation, contributes to the liberation of signaling helix-f. The complex after the bond breaking event is described by the 5c NO-HNOX system. The 5c NO-HNOX system undergoes significant structural transitions almost similar to 6c NO-HNOX. In contrast, the O_2_-ligated HNOX did not experience any significant change and the system maintained its compactness throughout the simulation (Figure 9). The structural transitions of helix-f and loops α and β are in agreement with earlier experimental and theoretical studies which suggest they play a crucial role in the activation of the soluble guanylate cyclase enzyme, after binding of the nitric oxide axial ligand [29,30]. The strong interaction of residues S137, R139 in loop γ and R116 in loop α with the heme moiety possibly contribute to the overall dynamic changes associated with sGC activation. 

## 3. Materials and Methods

### 3.1. Comparative Modeling of Human sGC HNOX Domain

To predict the structure of the HNOX domain, Protein BLAST [31] was used to find suitable protein structure templates. The 186 N-terminal residues that encode the HNOX domain of the sGC β subunit (NCBI Reference Sequence: NP_000848.1) [32], were subjected to comparative modeling. The structure of the nostoc bacterial sGC HNOX domain (PDB ID: 2O09) [6] was used as a template for the human sGC HNOX domain, with which it shares 34% identity. Homology modeling was done with Modeller9.16 [33], and the best model was selected based on the z-DOPE, estimated RMSD, estimated overlap scores and the quality of the Ramachandran plot [34]. Steric clashes were eliminated through energy minimization [35], and the structure was validated with MolProbity [36]. Furthermore, the builder module of pymol [37] was used to ligate NO, O_2_ and CO molecules to the heme group of the HNOX domain.

### 3.2. QM/MM Calculations

MCPB.py, a module of the Amber16 program package [38], which deals explicitly with metalloproteins [18], was employed to parametrize the H105-heme-NO/O_2_/CO active sites, acting as a bridge between Quantum mechanics (QM) and MD simulations. MCPB.py generates two models of which the smaller one is used for bond and angle parameters calculation and the larger one for charge calculation. The hybrid functional method B3LYP [39] with the 6-31G* basis set were applied for the optimization of the smaller model (heme-Fe-NO/O_2_/CO) while the force constant calculation was performed with the Seminario method [20]. This theoretical method uses a Cartesian Hessian matrix to calculate harmonic bond and angles. Atomic charges were estimated from the large model with the Merz-Kollman Restrained Electro Static Potential (RESP) method [40]. All calculations were performed using Gaussian09 [41], with the help of MCPB.py. 

### 3.3. MD Simulations

Five different complexes, apo-HNOX, 6c O_2_/NO/CO-HNOX and 5c NO-HNOX (where the Fe-His bond is broken) were explored with MD simulations. The calculated active site (H105-heme-NO/O_2_/CO) parameters were combined with the ff14SB force field to fully parametrize the protein systems [42]. The Amber16 tleap module was used to neutralize the system and add the missing hydrogen atoms. The protonation state of H105 was set manually depending on the presence of a bond between the imidazole ring and iron. The systems were immersed in orthogonal boxes with TIP3P-model water molecules [43]. All systems were simulated enabling periodic boundary conditions, and the Particle Mesh Ewald (PME) [44] method was employed to calculate long-range electrostatic interactions with a cutoff of 10 Å. Initially, the systems were minimized for 10,000 steps. During the subsequent annealing, the Langevin thermostat was operated to control the temperature from 0 to 300 K at constant volume (NVT) for 200 ps. The SHAKE algorithm was employed to constrain all bonds involving hydrogen atoms [45]. All systems were investigated for 30 ns at 300 K and 1 atm constant pressure (NPT). Trajectory snapshots were obtained every 2 fs. Basic root mean square deviation (RMSD), root mean square fluctuation (RMSF) and radius of gyration (R_g_) analysis was done to evaluate the systems’ stability throughout the simulation. Furthermore, hydrogen bond analysis and essential dynamics [46] were carried out with the CPPTRAJ module [20]. Visualization of the trajectories was achieved with VMD [47], while the plots were drawn with the help of Xmgrace [48] and Gnuplot [49].

### 3.4. Hydrogen Bond Occupancy

The analysis of hydrogen bonds is indispensable to studying the binding modes of complexes. For our systems, the hydrogen bonds of heme with the distal binding pocket residues of the HNOX domain, Y2, Y135, S137, E138, L142, R139, and the proximal binding pocket residues H105, R116, P118 were analyzed with CPPTRAJ. Additionally, solute-solute, solute-solvent, and solute-solvent-solute bridges hydrogen bond analysis of the entire HNOX systems was also carried out. The lifetime of hydrogen bond occupancies is indicative of the overall binding mode occupancies throughout the simulation and was visualized with Gnuplot.

### 3.5. Principal Component Analysis (PCA)

Essential dynamics were carried out to evaluate and interpret the multivariate data after detecting all correlated variable clusters. Furthermore, these correlated variables were converted to a small number of more meaningful uncorrelated variables [46]. The CPPTRAJ module of Amber16 was used to strip the solvent and ions from the trajectories before PCA analysis. The trajectories snapshots were then aligned over the average minimized structures. PCA was applied to all backbone atoms of the HNOX-O_2_/NO/CO and apo-HNOX systems. Diagonalization of covariance matrices assists in calculating the principal components of motion [50]. The trajectories were projected over eigenvectors corresponding to the first three largest eigenvalues of the correlation matrix. The most prominent fluctuations of different modes were investigated with the NMWiz VMD plugin [51].

## 4. Conclusions

Soluble guanylate cyclase is a regulatory protein involved in a multitude of physiological processes in humans such as platelet aggregation, vasodilation, and neurotransmission. Deciphering the ways in which it is regulated and regulates other proteins requires detailed structural characterization. The HNOX domain is the starting point in the signal transduction mechanism of this heterodimeric, multidomain protein. In the current study, we analyzed the effect of the physiologically relevant diatomic gaseous ligands CO/NO/O_2_ on the dynamics of the human HNOX domain, by employing a wide range of computational techniques. Residues in helix-f and loops α and β exhibited the most variable behavior, upon binding of the different gaseous ligands, highlighting the functional importance of these regions. These regions are located on the periphery of the inter-domain interface (HNOX-PAS) suggesting that they are involved in sGC activation. Among all complexes, 5c NO-HNOX experienced the highest positional displacement in helix-f and loop α, potentially facilitating the communication with adjacent sGC domains. The 5c NO-HNOX and 6c CO-HNOX residue R116 exhibits robust hydrogen bond interactions with the heme moiety, which could be critical to the communication with the neighboring domain. Interestingly, these interactions were either weak or absent in 6c NO-HNOX and 6c O_2_-HNOX. This work highlights important residues for sGC activation under physiological conditions that were not identified in our previous work [52] on the binding of sGC drugs. It is thus possible that a new class of compounds targeting the Fe-heme interface can be explored as potential sGC modulators. Exploring the HNOX binding modes is an important step in understanding the sGC activation mechanism and ultimately revealing regions with potential as drug targets for various sGC-associated diseases. 

## Figures and Tables

**Table 1:** Comparison of the apo-HNOX and HNOX-NO/O_2_/CO-ligated complexes (5c, 6c heme coordination states) hydrogen bond interactions of the heme propionic moiety with binding pocket residues.

Complexes	Acceptor	Donor	Percent of Frames of 30 ns Simulation Showing the Hydrogen Bond	Average Distance	Average Angle
Apo-HNOX	HM-187@O	R139@N	96.86	2.79	155.98
	HM-187@O	S137@OH	94.79	2.72	156.46
	HM-187@O	Y135@OH	87.41	2.70	158.41
	HM-187@O	Y2@N	44.39	2.84	153.42
	HM-187@O	R116@N	28.94	2.86	159.21
	HM-187@O	Y112@OH	2.06	2.76	164.44
5c HNOX-NO	HM-187@O	R116@N	54.75	2.92	145.26
	HM-187@O	S137@OH	35.37	2.81	150.52
	HM-187@O	R139@N	30.23	2.90	149.28
	HM-187@O	L142@N	4.74	2.90	155.25
	HM-187@O	Y49@OH	0.82	2.85	147.41
	HM-187@O	Y135@OH	0.18	2.84	148.21
	HM-187@O	E138@N	0.07	2.89	141.79
6c HNOX-NO	HM-187@O	R139@N	96.62	2.89	145.54
	HM-187@O	S137@OH	72.48	2.85	150.51
	HM-187@O	Y135@OH	40.07	2.85	150.70
	HM-187@O	R116@N	9.33	2.91	146.68
	HM-187@O	A117@N	3.26	2.88	142.18
	HM-187@O	E138@N	1.30	2.93	155.31
6c HNOX-O_2_	HM-187@O	R139@N	93.06	2.82	152.35
	HM-187@O	S137@OH	91.08	2.73	156.89
	HM-187@O	Y135@OH	87.66	2.69	160.04
	HM-187@O	Y2@N	17.57	2.82	153.32
	HM-187@O	R116@N	8.72	2.85	156.24
	HM-187@O	Y112@OH	1.34	2.73	165.64
6c HNOX-CO	HM-187@O	R116@N	90.82	2.79	155.25
	HM-187@O	R139@N	89.55	2.74	154.96
	HM-187@O	S137@OH	82.14	2.64	165.79
	HM-187@O	Y135@OH	8.11	2.65	165.85
	HM-187@O	Y112@OH	3.66	2.56	159.99
	HM-187@O	E138@N	1.46	2.85	158.29
	HM-187@O	M1@N	1.19	2.78	143.47
	HM-187@O	Y2@N	0.34	2.85	156.35

**Table 2:** Hydrogen bond occupancy of the histidine imidazole ring in the 5c NO-HNOX system.

Acceptor	Donor	Frames	Fraction	Distance Å	Angle
L101@O	H105@N	63780	0.8283	2.7872	163.1623
H105@O	A109@N	39130	0.5082	2.8682	158.5987
H105@O	L108@N	4746	0.0616	2.9051	150.2395
P118@O	H105@N	2749	0.0357	2.8107	155.5122
D102@O	H105@N	4722	0.0614	2.8373	151.0958
